# PPFIA1 expression associates with poor response to endocrine treatment in luminal breast cancer

**DOI:** 10.1186/s12885-020-06939-6

**Published:** 2020-05-14

**Authors:** Lutfi H. Alfarsi, Rokaya El Ansari, Madeleine L. Craze, Brendah K. Masisi, Ian O. Ellis, Emad A. Rakha, Andrew R. Green

**Affiliations:** 1 Nottingham Breast Cancer Research Centre, Division of Cancer and Stem Cells, School of Medicine, University of Nottingham Biodiscovery Institute, University Park, Nottingham, NG7 2RD UK; 2 Cellular Pathology, Nottingham University Hospitals NHS Trust, Hucknall Road, Nottingham, NG5 1PB UK

**Keywords:** Breast cancer, Oestrogen receptor, Endocrine resistance, PPFIA1, Liprin, Predictive biomarker

## Abstract

**Background:**

PPFIA1 is an important regulator of cell migration and invasion, regulating focal adhesion signalling and disassembly. PPFIA1 is frequently amplified in breast cancer, and recent functional studies indicate that PPFIA1 is an important promoter of migration and invasion in breast cancer. This study aims to evaluate the utility of PPFIA1 expression in the luminal breast cancer as a prognostic marker to predict the response to endocrine therapy.

**Methods:**

Large, well-characterised cohorts of primary luminal breast cancer patients with long-term follow-up was assessed for the clinical impact of PPFIA1 expression at the transcriptomic and proteomic levels. Prognostic significance of PPFIA1 and its relationship with clinical outcome and benefit of endocrine therapy were analysed. In addition, its association with other related-genes was analysed.

**Results:**

There was significant association between PPFIA1 expression and a member of the liprin family that involves in cell invasion (*PPFIBPI*), and the cell cycle regulator (*CCND1*), whereas a negative association was observed with the tumour suppressor gene (*CD82*). Patients with high PPFIA1 expression were associated with high risk of recurrence, distant metastasis and death from breast cancer (*P* < 0.05). Importantly, high PPFIA1 expression predicted relapse in a subset of patients who were subject to endocrine treatment alone, and was an independent prognostic marker of unfavourable outcome in these patients (*P* < 0.05).

**Conclusions:**

These findings support the proposed role for PPFIA1 as a regulator of cell migration in breast cancer and provides definitive evidence for the clinical utility of PPFIA1 expression in patients with luminal breast cancer. Most importantly, our data suggests that PPFIA1 might be a potential predictive marker for poor benefit from endocrine therapy.

## Background

Breast cancer exhibits significant heterogeneity with different molecular subtypes, and the main subtype of the disease are luminal, Estrogen Receptor positive (ER+)/HER2-negative, tumours which account approximately 75% of all breast cancers. This subtype remains heterogeneous in terms of prognosis and response to treatment [1, 2]. Endocrine therapy remains the key treatment for luminal breast cancer, whereas a small proportion of these tumours that are associated with high risk are offered chemotherapy. Although this treatment can potentially reduce the mortality rate, patients with similar prognostic factors at diagnosis can vary substantially in their response to treatment, develop resistance and later relapse [3, 4]. Therefore, identifying reliable predictive biomarkers for endocrine therapy benefit is required to stratify patients with luminal tumours for alternative therapy.

PTPRF Interacting Protein Alpha 1 (PPFIA1), also known as liprin-α1, belongs to the liprin family that includes liprin-α and liprin-β proteins [5]. PPFIA1 can interact directly with its several partners, and might form homo- or hetero-dimers with liprin-β proteins. PPFIA1 was originally found to control synapse formation and function in neuronal cells [6], whereas in non-neuronal cells it has been implicated in the regulation of cell motility [7]. Tumour cell migration and extracellular matrix degradation is required for tumour cells to form tumour metastasis [8], and recent studies suggest that PPFIA1 is an important promoter of tumour cells spreading in the extracellular matrix and is required for migration and invasion of tumour cells including breast cancer [9, 10]. PPFIA1 is located at the 11q13 amplification region, and is amplified in about 20% of breast cancer [11].

In this study, we hypothesised that PPFIAI will play a role in breast cancer and can provide important prognostic information that may help to risk stratify luminal tumors with respect to endocrine therapy.

PPFIA1 expression was assessed at the transcriptomic and proteomic levels utilising large and well-characterised annotated cohorts of luminal breast cancer.

## Methods

### *PPFIA1 mRNA* expression

PPFIA1 transcriptomic expression in luminal breast cancer and its role in predicting response to endocrine treatment was assessed in; The Molecular Taxonomy of Breast Cancer International Consortium (METABRIC) [12], Kaplan Meier Plotter-Breast Cancer (KM-Plotter) dataset [13] and Breast Cancer Gene-Expression Miner v4.3 (bc-GenExMiner v4.3) [14]. Additionally, the correlation of *PPFIA1* with other related-genes was investigated. The METABRIC cohort characteristics are in Supplementary Table 1.

### Protein expression cohort

PPFIA1 protein expression was assessed in a well-characterised series of luminal primary invasive breast cancer patients, with long-term follow-up (*n* = 521). Patients were presented at Nottingham City Hospital (1989–2006), as previously described [15]. The cohort characteristics are summarised in Supplementary Table 1.

### Evaluation of PPFIA1 protein expression

**Fig. 1:**
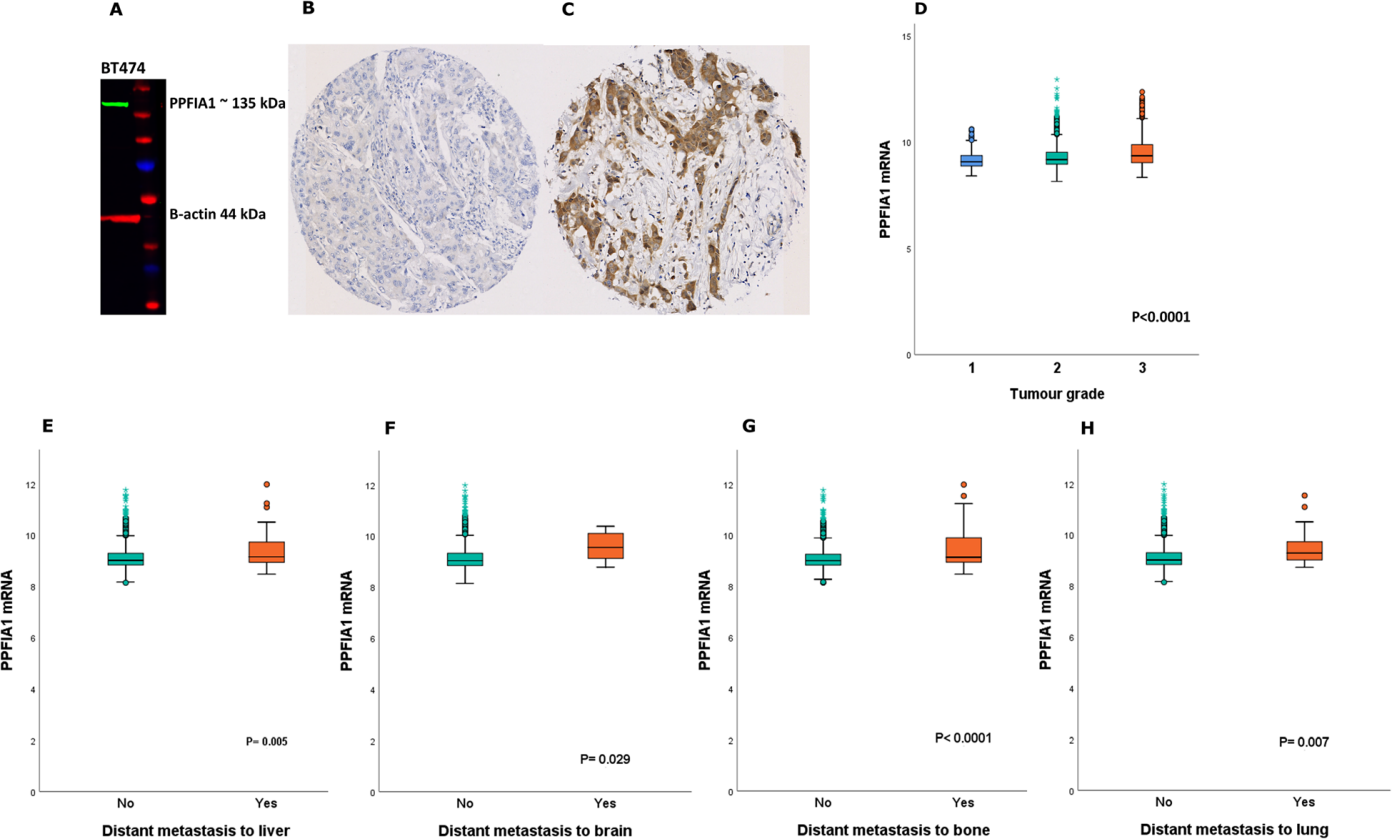
**a** Western blotting result for PPFIA1 expression in BT474 breast cancer cell lysate. Full-length blot is presented in Supplementary Figure 3. Representative immunostaining images of invasive breast cancer using IHC **b**) negative and **c**) positive for PPFIA1 protein expression. Association of *PPFIA1 mRNA* expression with **d**) tumour grade. The difference in *PPFIA1 mRNA* expression between cases who either developed distant metastasis to the **e**) liver **f**) Brain **g**) Bone and **h**) Lung or not, using the METABRIC cohort

The specificity of the PPFIA1 antibody (1500; A10388, ABclonal, UK) was validated by Western blotting using BT474 human breast cancer cell lysate (American Type Culture Collection; Rockville, MD, USA), which showed a single band of approximately the predicted size (135 kDa) (Fig. 1a).

PPFIA1 protein expression was evaluated using immunohistochemistry (IHC) on 4-μm tissue microarray (TMA) sections using Novolink polymer detection system (RE7150-K, Leica Biosystems, UK), as previously described using the PPFIA1 antibody at 1:100 dilution (A10388, ABclonal, UK) [15]. Evaluation of cytoplasmic staining for PPFIA1 in invasive tumour cells was performed using the modified histochemical score (H-score) [16]. TMA cores were only assessed if invasive tumour burden was > 15%. The PR positivity was defined as ≥ 1% staining.

### Statistical analysis

SPSS (version 25, Chicago, IL, USA) was used for all statistical analysis. The Chi-square test was used to evaluate the association between PPFIA1 *mRNA*/protein expression and clinicopathological parameters. To test correlation between two continuous normalised data, Pearson’s correlation coefficient was used. For the continuous variables, differences in mean between three or more groups were assessed using one-way analysis of variance (ANOVA) with the post-hoc Tukey multiple comparison test. T-test was used to assess the difference between two groups. Kaplan-Meier survival curves were used to investigate the association of PPFIA1 *mRNA*/protein expression with clinical outcome. The endpoint outcomes were breast cancer, recurrence and distant metastasis free survival. Cox regression analysis was used to evaluate the independent prognostic significance of PPFIA1 *mRNA*/protein expression. The Benjamini–Hochberg procedure for multiple test correction was used. Dichotomisation of PPFIA1 *mRNA*/protein expression into groups was determined using X-Tile (X-Tile Bioinformatics Software, Yale University, version 3.6.1). A *p* value of <0.05 was considered significant.

## Results

### PPFIA1 expression

Protein expression of PPFIA1 was observed in the cytoplasm of luminal tumours, and a representative images of IHC staining are shown in (Fig. 1b and c). High PPFIA1 protein expression (> 15 H-score) was observed in 394/521 (76%) of cases in luminal tumours, while high expression of *PPFIA1 mRNA* was observed in 1129/1398 (81%) of cases in the METABRIC cohort.

High *PPFIA1 mRNA* expression was associated with high tumour grade and the development of distant metastasis to the liver, brain, bone and lung (*P* < 0.05; Fig. 1d-h). However, the association between PPFIA1 protein expression with other clinicopathological parameters did not reach statistical significance, Supplementary Table 2.

### Prognostic value of PPFIA1 expression

**Fig. 2:**
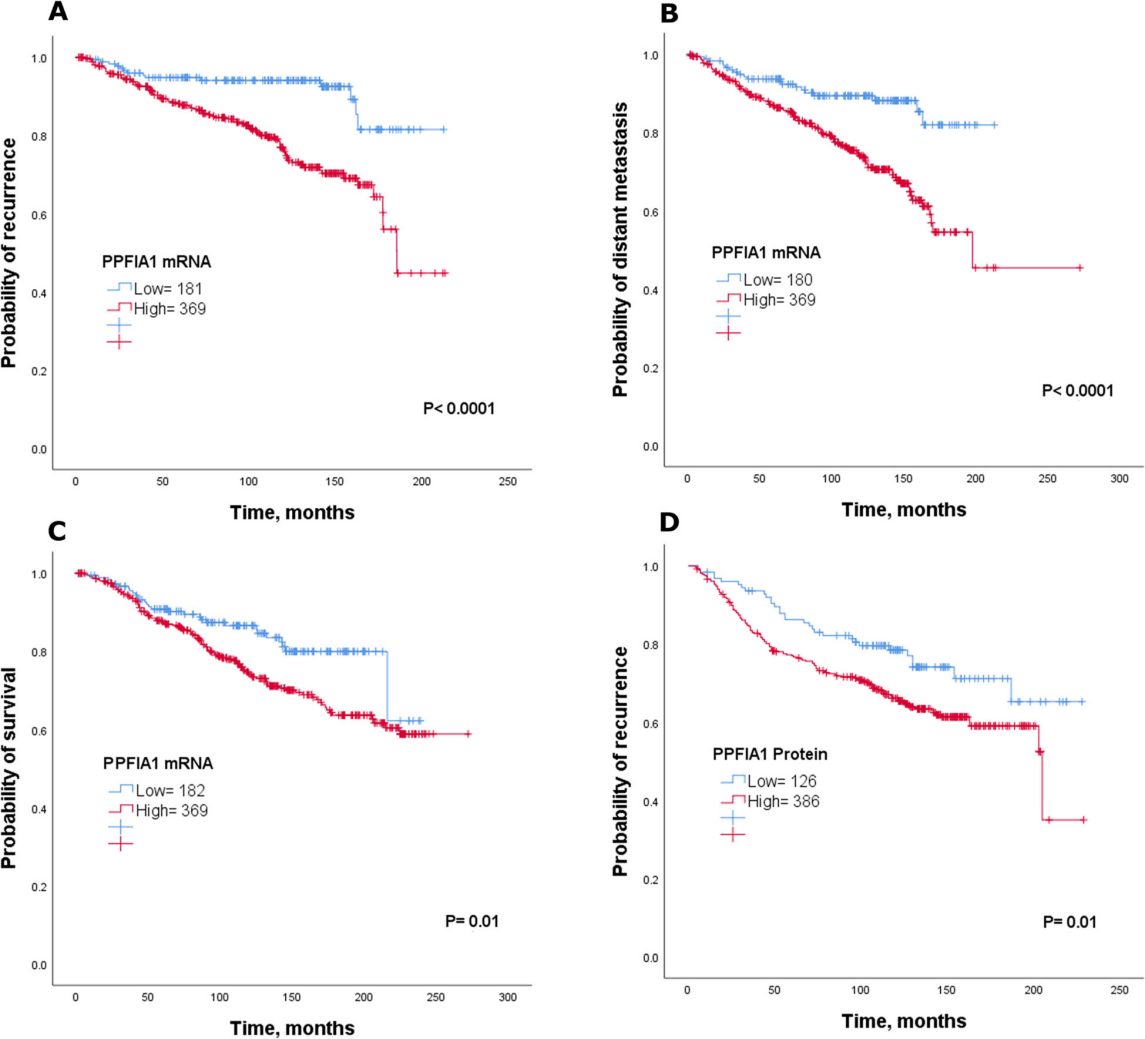
Kaplan–Meier of *PPFIA1 mRNA* and patient outcome in luminal breast cancer using the METABRIC cohort **a**) survival **b**) recurrence and **c**) distant metastasis. Kaplan–Meier of PPFIA1 protein and risk of **d**) recurrence

In the METABRIC cohort, patients showing high expression of PPFIA1 were statistically associated to shorter survival (*P* = 0.01; Fig. 2a) in compared with those showing low expression, which showed better clinical outcome. This association was confirmed using the KM-Plotter and bc-GenExMiner v4.3 datasets (*P* < 0.0001; Supplementary Figure 1A and B).

Likewise, results using the METABRIC cohort revealed that patients with tumours that highly expressed *PPFIA1 mRNA* significantly associated with poor recurrence and distant metastasis than those with low PPFIA1 expression (*P* < 0.05; Fig. 2b and c). These results were further validated using KM-Plotter and bc-GenExMiner v4.3 datasets, where higher levels of *PPFIA1 mRNA* were correlated to unfavourable outcome (*P* < 0.0001; Supplementary Figure 1C-E). This is consistent with what we found in the analysis of protein expression, whereby patients with high PPFIA1 protein expression had worse recurrence free survival compared to those with low PPFIA1 expression (*P* = 0.01; Fig. 2d).

### PPFIA1 expression predicts poor response to endocrine therapy

**Fig. 3:**
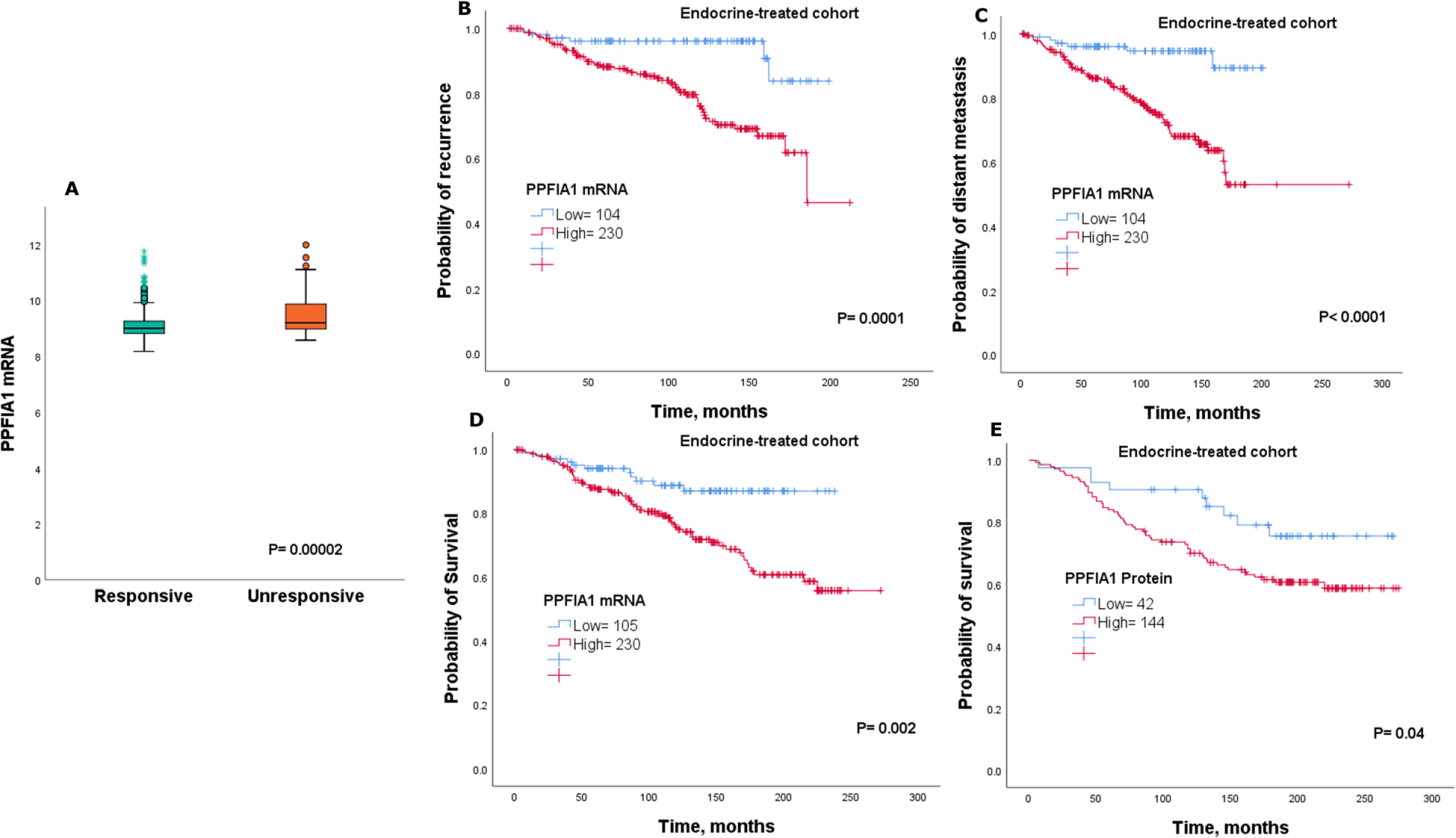
**a** Shows the difference in *PPFIA mRNA* expression between responsive and unresponsive cases to endocrine treatment using METABRIC cohort. Kaplan-Meier of *PPFIA1 mRNA* expression in patients with luminal breast cancer who received endocrine treatment only in the METABRIC cohort **b**) recurrence **c**) distant metastasis and **d**) survival. Kaplan-Meier of PPFIA1 protein expression in patients with luminal breast cancer who received endocrine treatment only **e**) survival

We next compared the levels of *PPFIA1 mRNA* expression between patients who received endocrine treatment and relapsed (unresponsive) with patients who received endocrine treatment but did not relapse (responsive). Results showed that *PPFIA1 mRNA* was highly expressed in unresponsive patients in comparison to those who responded to adjuvant endocrine therapy (*P* < 0.0001; Fig. 3a).

In patients who were subject to endocrine treatment only, high *PPFIA1 mRNA* was found to be significantly correlated with shorter recurrence, distant metastasis and survival (*P* < 0.05; Fig. 3b-d). This finding was further validated using KM-Plotter dataset (*P* < 0.05; Supplementary Figure 2A-C). In confirmation, patients receiving endocrine therapy with high PPFIA1 protein expression had a worse clinical outcome than those with low PPFIA1 expression (*P* = 0.04; Fig. 3d).

To further investigate the independence of PPFIA1 as a potential marker for outcome, multivariate cox regression analysis was performed. Results demonstrated that *PPFIA1 mRNA* expression was a predictor of poor outcome (*P* < 0.05), Supplementary Table 3. Further analysis on a subgroup of patients who were subject to endocrine treatment alone, demonstrated that *PPFIA1 mRNA* trend to be significant marker for relapse (*P* = 0.05), Supplementary Table 3. However, PPFIA1 protein expression, still an independent prognostic marker for clinical outcome in those patients receiving endocrine treatment (*P* < 0.05), Table 1.

**Table 1:** Multivariate cox analysis of associations between PPFIA1 protein expression and clinicopathological parameters in luminal breast cancer

Luminal breast cancer cohort			
	**Recurrence free survival**		
	HR (95% CI)	***P***	P*
PPFIA1	1.7 (1.1–2.6)	0.004	0.01
Tumour size	1.4 (1.1–1.9)	0.031	0.038
Tumour grade	1.3 (1.1–1.6)	0.017	0.02
Nodal stage	1.4 (1.1–1.8)	0.001	0.005
	**Distant metastasis free survival**		
	HR (95% CI)	***P***	P*
PPFIA1	1.4 (0.9–2.1)	0.05	0.06
Tumour size	1.7 (1.2–2.4)	0.001	0.002
Tumour grade	1.4 (1.1–1.8)	0.003	0.005
Nodal stage	1.5 (1.1–1.9)	0.0007	0.003
	**Breast cancer specific survival**		
	HR (95% CI)	***P***	P*
PPFIA1	1.6 (1.1–2.6)	0.02	0.025
Tumour size	1.8 (1.3–2.7)	0.0008	0.001
Tumour grade	1.7 (1.3–2.3)	0.00004	0.0002
Nodal stage	1.5 (1.2–2.0)	0.0007	0.001
Endocrine-treated cohort			
	**Recurrence free survival**		
	HR (95% CI)	***P***	P*
PPFIA1	2.5 (1.3–5.0)	0.006	0.01
Tumour size	2.0 (1.2–3.5)	0.0053	0.01
Tumour grade	1.5 (1.0–2.3)	0.048	0.06
Nodal stage	1.7 (1.1–2.4)	0.0051	0.02
	**Distant metastasis free survival**		
	HR (95% CI)	***P***	P*
PPFIA1	2.4 (1.2–4.7)	0.009	0.01
Tumour size	1.9 (1.1–3.2)	0.01	0.012
Tumour grade	2.0 (1.2–3.1)	0.002	0.005
Nodal stage	1.8 (1.2–2.6)	0.001	0.005
	**Breast cancer specific survival**		
	HR (95% CI)	***P***	P*
PPFIA1	2.8 (1.3–5.8)	0.005	0.008
Tumour size	2.4 (1.3–4.1)	0.002	0.005
Tumour grade	2.6 (1.6–4.4)	0.0001	0.0005
Nodal stage	1.6 (1.1–2.5)	0.009	0.01

P*: Adjusted *p*-value

### PPFIA1 expression and other associated genes

To further understand the potential roles of PPFIA1 in luminal breast cancer and how it might affect the response to the endocrine therapy, correlation of *PPFIA1 mRNA* expression with other associated-genes was investigated using the METABRIC cohort, and validated using bc-GenExMiner v4.3 dataset, Supplementary Table 4. The selection of these genes was based on previous publications, in which interact with PPFIA1 or support its biological function [17–21]. Results showed a positive correlation between *PPFIA1 mRNA* expression and PPFIBPI, also known as (liprin-β1) which is member of the liprin family that involves in cell invasion. Further, a positive correlation was observed with the well-known cell cycle regulators (*CCND1*, *CCNA2* and *CCNB1*). On the other hand, a negative correlation was observed between *PPFIA1 mRNA* expression and integrin family genes (*ITGB1* and *ITGA5*) and the tumour suppressor gene (*CD82*). Taken together, these findings suggest a key role of PPFIA1 in the invasion of luminal tumours and response to treatment.

## Discussion

This study has explored the potential effects of PPFIA1 expression in luminal tumours. Our findings establish an important role for PPFIA1 expression in luminal breast cancer and response to endocrine therapy. This study of a well-characterised cohort of clinically annotated patients with early luminal breast cancer demonstrates that high PPFIA1 expression associates with aggressive clinicopathological parameters. Our study also confirmed that patients with high PPFIA1 expression had unfavourable clinical outcome and poor response to endocrine therapy.

PPFIA1 is part of a multi-protein complex that is important in the regulation of tumour cell migration and invasion [7, 10]. High expression of PPFIA1 has been reported in various cancers including breast cancer [9, 11, 18, 22]. In support of these findings, we found a proportion of breast cancers with high *mRNA* and protein expression. In addition, we found that PPFIA1 expression was associated with aggressive clinicopathological parameters. Previous studies show a contradictory role of PPFIA1 in regards to the invasiveness of tumour cells, where silencing of PPFIA1 affects the regulation of dynamic events associated with tumour cells invasion [9, 17] whereas the effect is opposite in head and neck squamous cell carcinoma [23]. However, our finding confirmed an oncogenic role of PPFIA1 in tumour cell invasion where we found high PPFIA1 levels is significantly correlated with the development of distant metastasis in the liver. This result ties well with previous study, which showed PPFIA1 expression was significantly higher in liver metastatic tumours than in the primary tumours [24], and PPFIA1 overexpression enhanced cell spreading and cell migration [7]. Altogether, these findings suggesting a potential role for PPFIA1 expression in the invasive process within breast tumour cells.

Another important finding of this study is that high PPFIA1 expression is associated with short survival within luminal breast cancer subtype. This is consistent with a previous study where association of PPFIA1 amplification with poor survival was only observed in the subset of ER+ cancers with no prognostic effect on ER negative or HER2+ breast cancer subtypes [18]. Indeed, our results demonstrate the clinical role of PPFIA1 expression at both transcriptomic and proteomic levels in predicting the recurrence and distant metastasis within luminal breast cancer. These findings suggest critical roles of PPFIA1 in luminal breast cancer, and could be used as a marker of poor prognosis.

Despite endocrine treatment has proven its enormous value in the treatment of luminal breast cancer, resistance stills a major issue in a proportion of these patients. Therefore, it remains necessary to predict which patients who will potentially benefit or resist this treatment through identifying valuable clinical biomarkers to guide choices of therapies [25]. ER and Progesterone Receptor (PR) still the only used as a guide to response of endocrine treatment in clinic, despite the increase and widespread of the advanced technology [26]. Multigene signatures, such as Oncotype DX, EndoPredict and Mammaprint can be used as additional prognostic tools for risk stratification. However, these tools are unable to predict patients’ benefit from anti-oestrogen treatment in early stage, and in cases with intermediate score which might lead to an inconclusive prognosis [25, 27]. In this study, we show that patients with luminal tumours and higher expression of PPFIA1 are less likely to benefit from endocrine therapy. High PPFIA1 expression predicted higher probability of relapse in patients who were given endocrine treatment. These findings suggest that PPFIA1 expression could provide complementary information to currently existing ER expression to predict failure of endocrine treatment, and could easily be routinely measured by the conventional IHC. However, further studies of pre-clinical and clinical are required to confirm and validate the clinical value of PPFI1 as predictive marker, particularly as the exact relationship between PPFIA1 and hormonal signals remains unclear.

*PPFIA1* is located at the 11q13 amplification region, which occurs in about 20% of breast tumours [11]. *CCND1* is the important driver gene of the 11q13 amplicon, and *PPFIA1* amplification was found in all *CCND1*-amplified breast cancers [11, 18]. We have demonstrated that *PPFIA1 mRNA* was positively correlated with *CCND1 mRNA* levels in cases with luminal tumours. Previous studies found that high *CCND1* expression and amplification associates with an impaired response to tamoxifen treatment and high risk of relapse in luminal breast cancer [28, 29]. Taken together, these findings might suggest a role on how PPFIA1 expression associates with poor response to endocrine therapy.

Previous studies suggested that integrin signalling might have a significant role in cell migration and invasion, and also in modulating resistance to anti-cancer treatments [30, 31]. In the current study, we found a correlation between *PPFIA1 mRNA* expression and integrin family members in luminal breast cancer. Our finding supports previous studies that reported PPFIA1 as an essential regulator of integrin signalling which required for efficient cell motility [20, 32]. Further, we found that high PPFIA1 expression is negatively associated with the tumour metastasis suppressor gene (*CD82*), which is suggested to inhibit cell migration and invasion via regulating the integrin-mediated signalling [33]. In breast tumours, CD82 reduces in vitro migration and in vivo metastasis [34]. In line with our finding, silencing of PPFIA1 was found to upregulate the CD82 cell surface protein in breast tumours [21]. Altogether, these findings suggest that PPFIA1 could potentially act as in regulating CD82 expression and integrin signalling causing tumour progression and invasion in luminal breast cancer.

PPFIBPI, also known as liprin-β1, is a member of liprin family, and is one of the many binding partners of PPFIA1. A previous study showed that silencing of PPFIBPI causes a significant decrease of cell migration and it was demonstrated that PPFIA1 and PPFIBPI may co-operate in the regulation of tumour cell migration [7]. In this study, a positive correlation was observed between *PPFIA1* and *PPFIBPI* in patients with luminal breast cancer, which may confirm their regulatory roles in the migration of tumour cells.

## Conclusions

This study provides evidence for the role of PPFIA1 expression in luminal breast cancer. Most importantly, our study has clearly showed that PPFIA1 expression is a potential marker for poor benefit from endocrine therapy and its measurement may guide the clinician for alternative therapy.

## Supplementary information

**Additional file 1: Supplementary Figure 1.** Kaplan–Meier of *PPFIA1 mRNA* and patient outcome in luminal breast cancer using KM-Plotter dataset for **A)** survival **C)** recurrence and **D)** distant metastasis, and using bc-GenExMiner v4.3 for **B)** survival and **E)** metastasis relapse.

**Additional file 2: Supplementary Figure 2.** Kaplan-Meier of *PPFIA1 mRNA* expression in patients with luminal breast cancer who received endocrine treatment only using KM-Plotter dataset **A)** recurrence **B)** distant metastasis and **C)** survival.

**Additional file 3: Supplementary Figure 3**

**Additional file 4: Supplementary Table 1.** Clinicopathological characteristics of luminal breast cancer cohorts.

**Additional file 5: Supplementary Table 2.** Association of PPFIA1 protein expression and clinicopathological parameters in Nottingham cohort.

**Additional file 6: Table 3.** Multivariate cox analysis of associations between *PPFIA1 mRNA* expression and clinicopathological parameters in luminal breast cancer using METABRIC cohort.

**Additional file 7: Supplementary Table 4.** Correlation of *PPFIA1 mRNA* expression with the expression of other related genes.

## Data Availability

The datasets used and/or analysed during the current study are available from the corresponding author on reasonable request.

## Abbreviations

ER +: Oestrogen receptor positive
HER2: Human epidermal growth factor receptor 2
NPI: Nottingham Prognostic Index
IHC: Immunohistochemistry
TMAs: Tissue microarrays
PR: Progesterone Receptor
