# Supplementary figures and images for: PPFIA1 expression associates with poor response to endocrine treatment in luminal breast cancer
Source: BMC Cancer. 2020 May 14;20:425. doi: 10.1186/s12885-020-06939-6 (PMC7227113; doi:10.1186/s12885-020-06939-6)

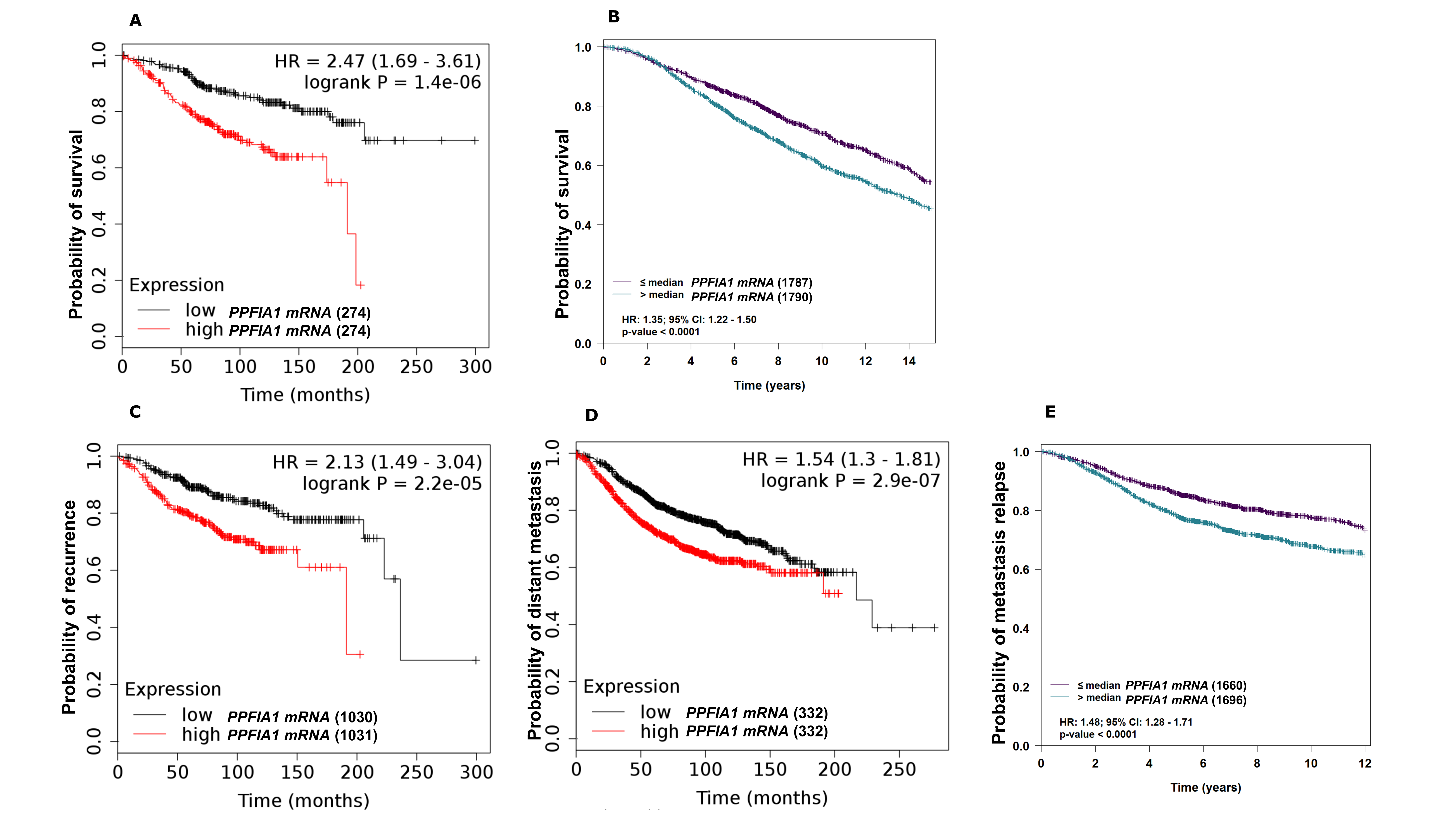

Supplement: Supplementary file 1 — Additional file 1: Supplementary Figure 1. Kaplan–Meier of PPFIA1 mRNA and patient outcome in luminal breast cancer using KM-Plotter dataset for A) survival C) recurrence and D) distant metastasis, and using bc-GenExMiner v4.3 for B) survival and E) metastasis relapse. [file 12885_2020_6939_MOESM1_ESM.tif]

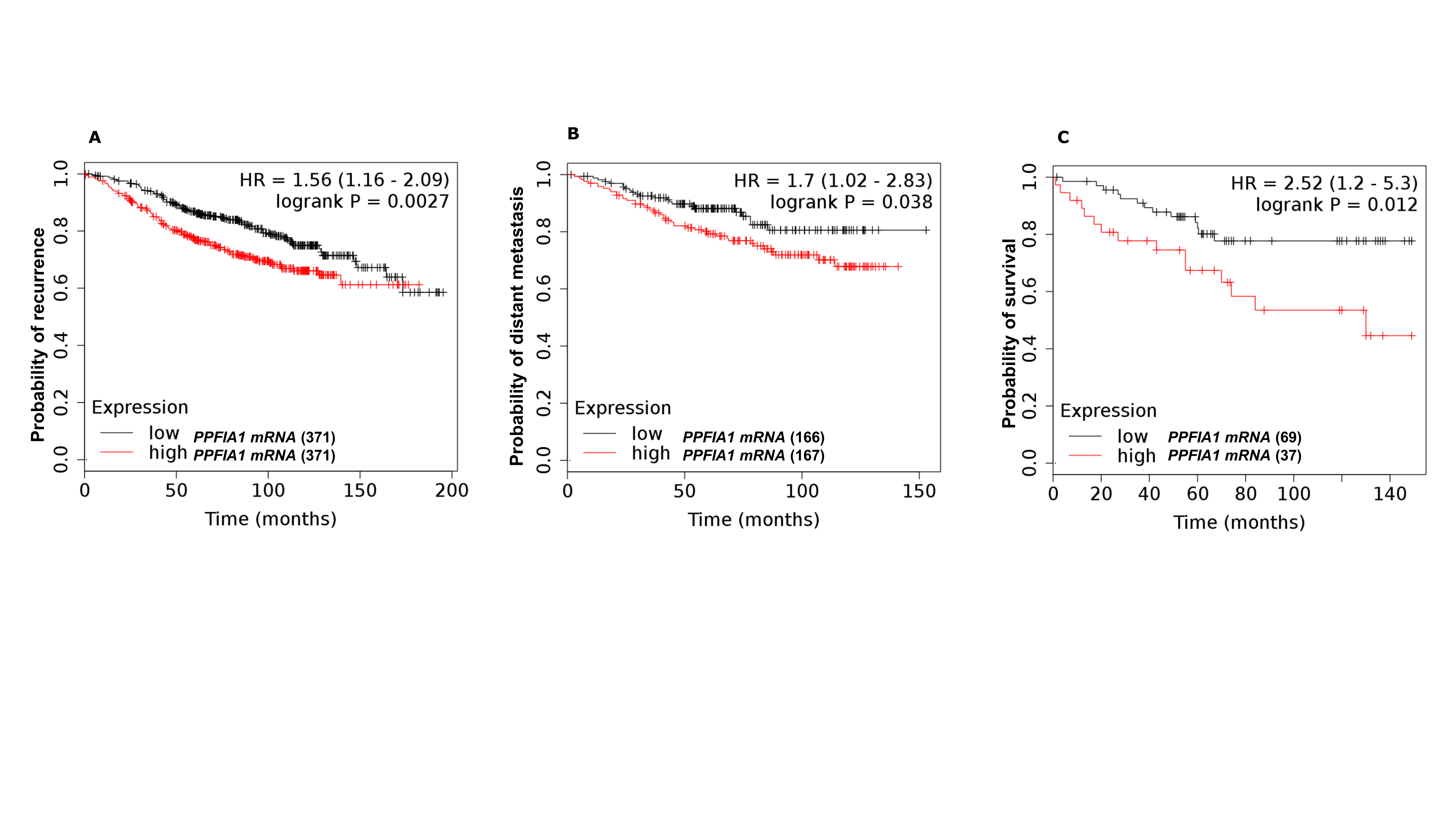

Supplement: Supplementary file 2 — Additional file 2: Supplementary Figure 2. Kaplan-Meier of PPFIA1 mRNA expression in patients with luminal breast cancer who received endocrine treatment only using KM-Plotter dataset A) recurrence B) distant metastasis and C) survival. [file 12885_2020_6939_MOESM2_ESM.tiff]

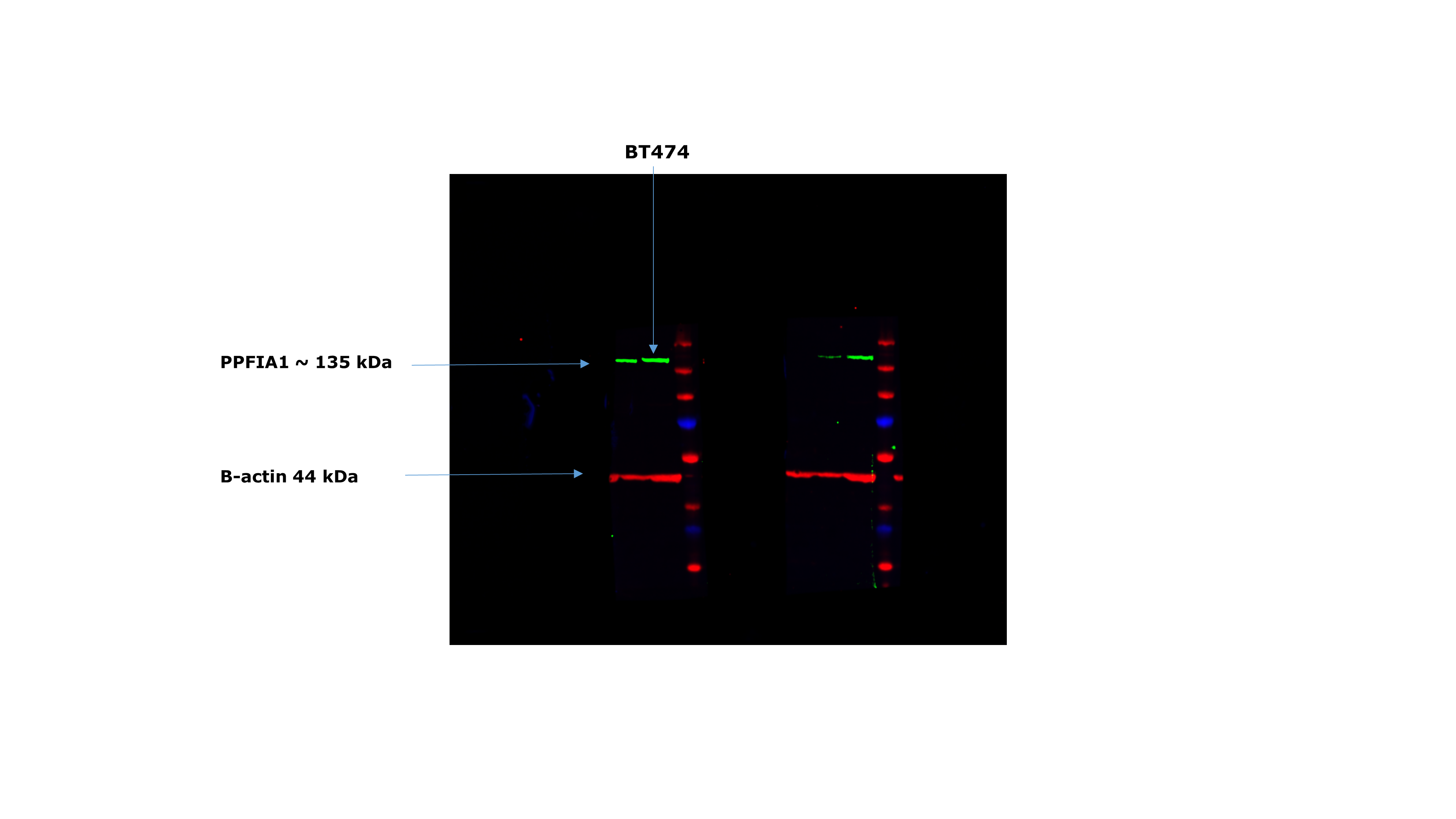

Supplement: Supplementary file 3 — Additional file 3: Supplementary Figure 3 [file 12885_2020_6939_MOESM3_ESM.tif]
