# Supplementary material for: PPFIA1 expression associates with poor response to endocrine treatment in luminal breast cancer
Source: BMC Cancer. 2020 May 14;20:425. doi: 10.1186/s12885-020-06939-6 (PMC7227113; doi:10.1186/s12885-020-06939-6)
Supplement: Supplementary file 4 — Additional file 4: Supplementary Table 1. Clinicopathological characteristics of luminal breast cancer cohorts. [file 12885_2020_6939_MOESM4_ESM.docx]

**Supplementary table 1:** Clinicopathological characteristics of luminal breast cancer cohorts.

| **Parameters** | **METABRIC cohort**  ***mRNA*** | **Nottingham cohort**  **Protein** |
| --- | --- | --- |
|  | No. (%) | No. (%) |
| ***Age***  *<50*  *≥50*  ***Tumor size (cm)***  *<2 cm*  *≥2 cm*  ***Tumour grade***  *1*  *2*  *3*  ***Nottingham Prognostic Index***  *GPG*  *MPG*  *PPG*  ***Nodal stage***  *1*  *2*  *3*  ***Vascular invasion***  *Negative*  *Positive*  ***Endocrine therapy alone***  *No*  *Yes*  *Other**  ***Recurrence***  *No*  *Yes*  *Unknown*  ***Distant metastasis***  *No*  *Yes*  *Unknown*  ***Progesterone receptor***  *Negative*  *Positive* | 228 (15)  1278 (85)  475 (31.5)  1031 (68.5)  166 (11.5)  707 (49.1)  565 (38.4)  623 (41.3)  772 (51.2)  111 (7.5)  404 (36.2)  634 (56.8)  78 (7)  N/A  234 (15.5)  384 (25.5)  888 (59)  496 (33)  118 (7)  892 (60)  476 (31)  143 (10)  887 (59)  486 (23.2)  1020 (76.8) | 449 (31)  1006 (69)  806 (55.7)  640 (44.3)  388 (24.7)  661 (42.1)  522 (33.2)  598 (41.4)  668 (46.1)  180 (12.5)  1025 (65.1)  439 (27.9)  111 (7)  1000 (69)  439 (31)  810 (50)  554 (35)  237 (15)  953 (60)  618 (38)  30 (2)  1079 (67)  514 (32)  8 (1)  300 (21.3)  1103 (78.7) |
| **GPG:** Good prognostic group**; MPG:** Moderate prognostic group**; PPG:** Poor prognostic group  * Include patients who received chemotherapy alone or combination of chemotherapy and endocrine therapy. | | |
