# Supplementary material for: PPFIA1 expression associates with poor response to endocrine treatment in luminal breast cancer
Source: BMC Cancer. 2020 May 14;20:425. doi: 10.1186/s12885-020-06939-6 (PMC7227113; doi:10.1186/s12885-020-06939-6)
Supplement: Supplementary file 5 — Additional file 5: Supplementary Table 2. Association of PPFIA1 protein expression and clinicopathological parameters in Nottingham cohort. [file 12885_2020_6939_MOESM5_ESM.docx]

**Supplementary table 2:** Association of PPFIA1 protein expression and clinicopathological parameters in Nottingham cohort.

|  | PPFIA1 expression | | *P* | *P** |
| --- | --- | --- | --- | --- |
|  | **Low**  No. (%) | **High**  No. (%) |  |  |
| Tumour size (cm)  <2 cm  ≥2 cm | 71 (24.5)  56 (24.6) | 219 (75.5)  172 (75.4) | 0.98 | 1.31 |
| Tumour grade  1  2  3 | 23 (24.5)  57 (24.6)  47 (24.5) | 71 (75.5)  175 (75.4)  145 (75.5) | 1.00 | 1.14 |
| NPI  GPG  MPG  PPG | 47 (23.9)  60 (23.3)  20 (31.3) | 150 (76.1)  197 (76.7)  44 (68.8) | 0.40 | 1.08 |
| Nodal stage  1  2  3 | 78 (23.7)  36 (24.2)  13 (32.5) | 251 (76.3)  113 (75.8)  27 (67.5) | 0.471 | 0.94 |
| Vascular invasion  Negative  Positive | 93 (24.7)  34 (19.1) | 247 (72.6)  144 (80.9) | 0.03 | 0.34 |
| Progesterone receptor  Negative  Positive | 33 (31.4)  94 (22.8) | 72 (68.6)  319 (77.2) | 0.06 | 0.26 |
| *P**: Adjusted P values  NPI: Nottingham Prognostic Index; GPG: Good prognostic group; MPG: Moderate prognostic group; PPG: Poor prognostic group | | | | |
