# Supplementary material for: PPFIA1 expression associates with poor response to endocrine treatment in luminal breast cancer
Source: BMC Cancer. 2020 May 14;20:425. doi: 10.1186/s12885-020-06939-6 (PMC7227113; doi:10.1186/s12885-020-06939-6)
Supplement: Supplementary file 6 — Additional file 6: Table 3. Multivariate cox analysis of associations between PPFIA1 mRNA expression and clinicopathological parameters in luminal breast cancer using METABRIC cohort. [file 12885_2020_6939_MOESM6_ESM.docx]

**Table 3:** Multivariate cox analysis of associations between *PPFIA1 mRNA* expression and clinicopathological parameters in luminal breast cancer using METABRIC cohort.

| *Luminal breast cancer cohort* | | | | |
| --- | --- | --- | --- | --- |
|  | **Recurrence free survival** | | | |
|  | HR (95% CI) | ***P*** | ***P**** | |
| *PPFIA1*  Tumour size  Tumour grade  Nodal stage | 0.3 (0.1-0.8)  1.3 (0.8-2.1)  1.3 (0.9-1.8)  1.1 (0.7-1.6) | 0.01  0.16  0.07  0.48 | 0.05  0.2  0.1  0.6 | |
|  | **Distant metastasis free survival** | | | |
|  | HR (95% CI) | ***P*** | | ***P**** |
| *PPFIA1*  Tumour size  Tumour grade  Nodal stage | 0.3 (0.1-0.8)  1.9 (1.1-3.2)  1.5 (1.0-2.2)  1.3 (0.9-2.0) | 0.019  0.014  0.02  0.08 | | 0.04  0.07  0.03  0.1 |
|  | **Breast cancer specific survival** | | | |
|  | HR (95% CI) | ***P*** | | ***P**** |
| *PPFIA1*  Tumour size  Tumour grade  Nodal stage | 0.4 (0.1-1.0)  1.9 (1.0-3.5)  2.0 (1.3-3.2)  1.4 (0.9-2.0) | 0.05  0.02  0.002  0.09 | | 0.08  0.05  0.01  0.1 |

***Endocrine-treated cohort***

|  | **Recurrence free survival** | | |
| --- | --- | --- | --- |
|  | HR (95% CI) | ***P*** | ***P**** |
| *PPFIA1*  Tumour size  Tumour grade  Nodal stage | 0.2 (0.7-0.7)  1.1 (0.6-1.9)  1.2 (0.8-1.8)  1.0 (0.7-1.6) | 0.01  0.64  0.32  0.67 | 0.05  0.8  1.06  0.8 |
|  | **Distant metastasis free survival** | | |
|  | HR (95% CI) | ***P*** | ***P**** |
| *PPFIA1*  Tumour size  Tumour grade  Nodal stage | 0.2 (.08-0.8)  1.4 (0.7-2.6)  1.4 (0.8-2.2)  1.4 (0.9-2.1) | 0.02  0.24  0.15  0.12 | 0.1  0.3  0.2  0.3 |
|  | **Breast cancer specific survival** | | |
|  | HR (95% CI) | ***P*** | ***P**** |
| *PPFIA1*  Tumour size  Tumour grade  Nodal stage | 0.1 (.04-0.8)  1.8 (0.9-3.7)  1.7 (0.9-3.0)  1.2 (0.8-2.0) | 0.04  0.08  0.05  0.29 | 0.2  0.1  0.1  0.3 |
| ***P*:*** Adjusted p-value | | |  |
