# Supplementary material for: PPFIA1 expression associates with poor response to endocrine treatment in luminal breast cancer
Source: BMC Cancer. 2020 May 14;20:425. doi: 10.1186/s12885-020-06939-6 (PMC7227113; doi:10.1186/s12885-020-06939-6)
Supplement: Supplementary file 7 — Additional file 7: Supplementary Table 4. Correlation of PPFIA1 mRNA expression with the expression of other related genes. [file 12885_2020_6939_MOESM7_ESM.docx]

**Supplementary table 4:** Correlation of *PPFIA1* *mRNA* expression with the expression of other related genes.

|  | METABRIC cohort | | BC-GeneMiner dataset | |
| --- | --- | --- | --- | --- |
|  | Correlation Coefficient | ***P*** | Correlation Coefficient | ***P*** |
| *PPFIBPI*  *CCND1*  *CCNA2*  *CCNB1*  *ITGB1*  *ITGA5*  *CD82* | 0.16  0.56  0.2  0.32  -0.21  0.1  -0.26 | 3.26e-10  1.33e-199  4.20e-14  1.21e-34  1.93e-16  0.00009  2.75e-23 | 0.08  0.47  0.23  0.25  -0.07  -0.02  -0.19 | <0.0001  <0.0001  <0.0001  <0.0001  <0.0001  0.04  <0.0001 |
